# Characterization of a Novel Lysozyme-Like 4 Gene in the Rat

**DOI:** 10.1371/journal.pone.0027659

**Published:** 2011-11-15

**Authors:** Ganapathy Narmadha, Katakam Muneswararao, Angireddy Rajesh, Suresh Yenugu

**Affiliations:** Department of Animal Sciences, University of Hyderabad, Hyderabad, India; National Institute of Immunology, India

## Abstract

Lysozyme-like proteins (LYZLs) belong to the class of c-type lysozymes and are not well characterized in many species including the rat. In this study, using *in silico* and molecular biology techniques, we report the identification, cloning and characterization of rat *Lyzl4* gene and also determine the expression pattern of *Lyzl1*, *Lyzl3* and *Lyzl6*. The rat *Lyzl* genes were found to be distributed on three chromosomes and all of them retained the characteristic eight cysteine signature of c-type lysozyme. Homology modeling of rat LYZL4 indicated that its structure is similar to that of the mouse SLLP1. In the male reproductive tract of rat, Lyzl gene expression was confined to the testis. *Lyzl1* and *Lyzl4* were found to be expressed in tissues beyond the male reproductive tract, whereas *Lyzl3* and *Lyzl6* were not. *Lyzl* expression in the developing (10–60 day old) rats was androgen dependent in the testis. Immunodetection using antibodies against rat LYZL4 revealed the presence of LYZL4 protein in the germinal layer of the testes and on the sperm tail. Recombinant LYZL4 did not exhibit antibacterial, muramidase and isopeptidase activities characteristic to c-type lysozyme. To the best of our knowledge, for the first time we report the characterization of *Lyzl* genes in the rat. Results of our study indicate that rat LYZL proteins may have an important role in male reproductive tract function.

## Introduction

In the 1930s Alexander Flemming discovered lysozyme (EC 3.2.17), a remarkable bactericidal agent [1]. Basing on their physical and functional properties, a wide variety of lysozymes have been identified. They are mainly classified into six families, namely, g-type (goose type), c-type (chicken-type), invertebrate type (I-type), phage, bacterial and plant [2]. Among them, the c-type are widely distributed across the species [3], [4], [5], [6] and in various organ systems including the male reproductive tract. C-type lysozymes are N-acetylglucosamine binding proteins and are of two types, namely, the non-calcium binding c-lysozymes and the calcium-binding c-lysozymes [7]. The enzymatic action of c-type lysozyme involves the hydrolysis of beta-1,4 glycosidic bonds between C-1 of N-acetylmuramic acid and C-4 of N-acetylglucosamine in the peptidoglycan of bacterial cell walls. Its ability to act on bacterial membranes confers the bactericidal activity and thereby has a role in innate immunity [3].

The male reproductive tract is a dynamic organ system involved in both endocrine and reproductive functions. Spermatozoa that emerge from the testis are immature, non-motile and lack fertilizing ability. Their passage through the epididymis allows interaction with a wide variety of epididymal secreted proteins resulting in acquisition of motility and fertilizing ability. Proteins secreted into the epididymal lumen [8] include defensins [9], [10], lipocalins [11], cathelicidins [12], members of the sperm associated antigen 11 family [13], protease inhibitors [14], [15], [16] and enzymes including the c-type lysozyme [17], [18].

In humans, besides the c-lysozyme, lysozyme like genes were identified [19] and some of them (*LYZL2, LYZL/SLLP1, LYZL4* and *LYZL6*) are found to be expressed in the male reproductive tract [17], [18]. Spermatozoa incubated with antibodies to human SLLP1 failed to fertilize eggs, thereby demonstrating a role in male reproductive function [18]. Similarly, incubation of spermatozoa with the mouse LYZL4 antibodies resulted in loss of fertilizing ability [20]. However, the role of other three mouse c-lysozymes in the reproductive tract is not yet clear. Unlike the human and mouse counter parts, the rat *Lyzl* genes are not characterized. In the rat genome available at GenBank, of the four c-type lysozymes (*Lyzl1*, *Lyzl3*, *Lyzl*4 and *Lyzl6*), *Lyzl*4 sequence is predicted, whereas the other three were annotated using the whole genome shot gun analyses. Except for their gene identification, the functional role is not reported till now.

In this study, we report the identification and characterization of the rat *Lyzl*4. Further, the expression profile of the *Lyzl* transcripts (*Lyzl1, Lyzl3, Lyzl4 and Lyzl6*) was analyzed and their androgen dependence determined. Their possible contribution to the male reproductive tract immunity was analyzed.

## Results

### *In silico* analyses

**Figure 1:**
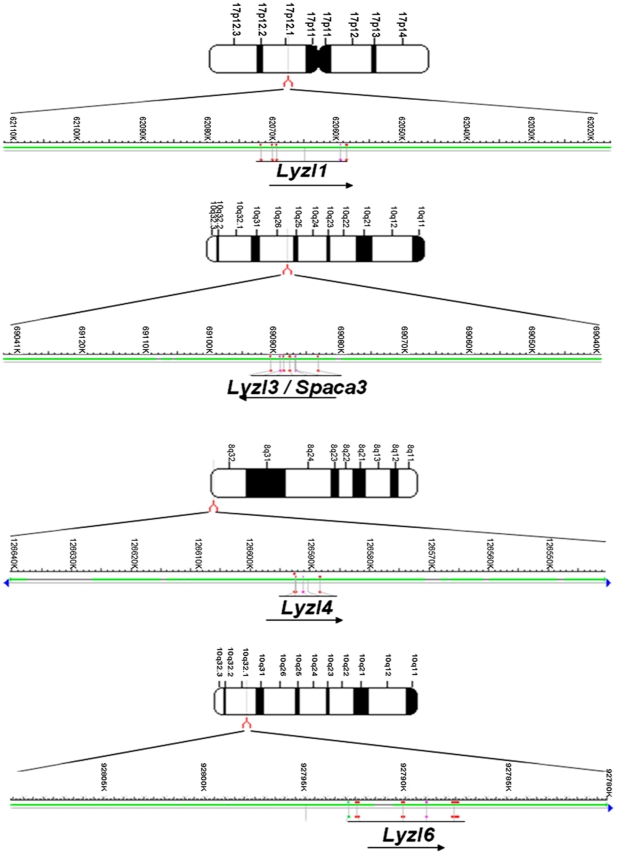
Rat *Lyzl* gene localization. Arrows indicate direction of transcription. Positions were taken from the Mapview (RGSC v3.4) at the National Center for Biotechnology Information (NCBI) website.

**Figure 2:**
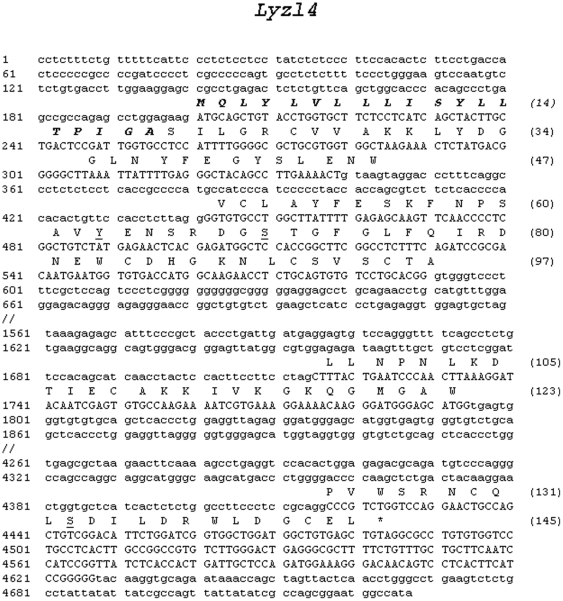
Rat chromosomal sequence aligned with *Lyzl4* mRNA and predicted amino acid sequence. Exons are in upper case letters and introns in lower case. Amino acids are indicated in single letters. Numbers in parentheses indicate amino acids of the protein. The gene sequence was extracted from GenBank NW_047803.1. The rat *Lyzl4* cDNA sequence was submitted to Genbank and was assigned the accession number HM125534. Predicted signal peptide cleavage site is indicated in bold italics. Posttranslational modification sites are indicated: single underlined – phosphorylation.

**Figure 3:**
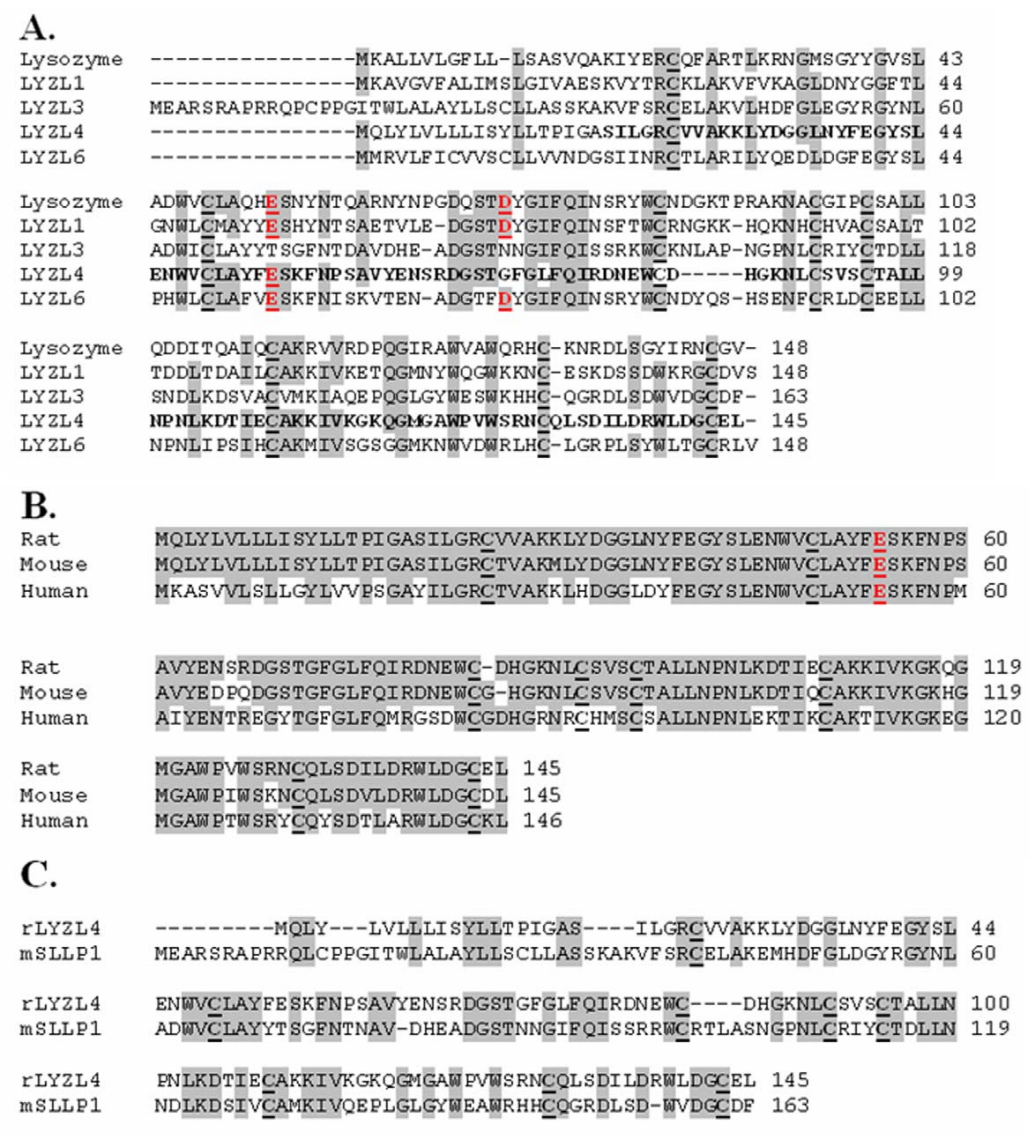
Multiple sequence alignment of LYZL proteins. **A**) Rat LYZL proteins. **B**) Alignment of rat, mouse and human LYZL4 protein sequences. The conserved amino acid residues are shaded. Amino acids in the active site responsible for the enzyme activity are shown in red. The eight cysteines of the c-type lysozyme signature are indicated in bold and underlined. The LYZL4 sequence shown in bold was expressed as a recombinant protein. C) Alignment of rat LYZL4 and mouse SLLP1.

**Figure 4:**
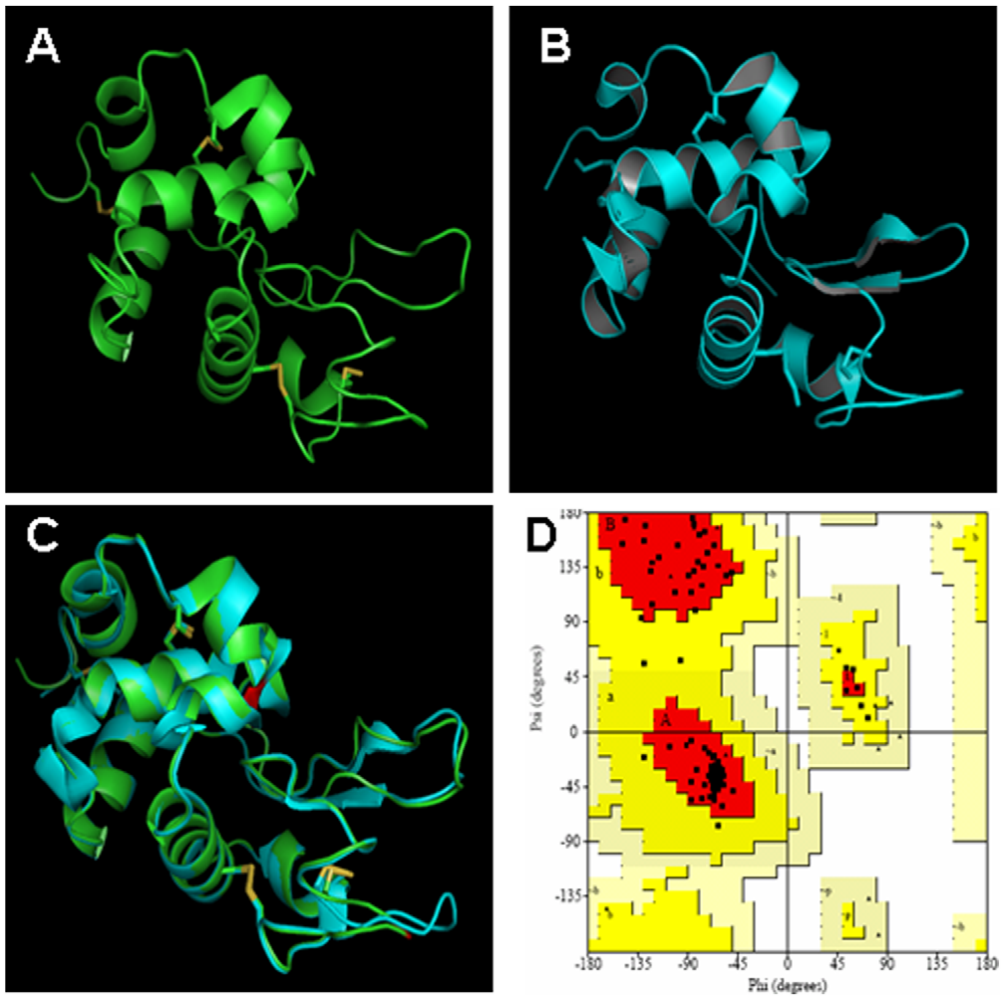
Homology modeling of rat LYZL4. **A**) Cartoon model of rat LYZL4. Disulfide bonds are indicated in yellow. **B**) Mouse SLLP1 protein model used as template. **C**) Rat LYL4 and mouse SLLP1 cartoon superimposition. **D**) Ramachandran plot for the rat LYZL4.

Using gene specific primers, rat *Lyzl4* mRNA transcript was amplified and sequenced. It is located on chromosome 8, whereas *Lyzl1*, *Lyzl3* and *Lyzl6* are present on chromosome 10 and 17 (Figure 1). The *Lyzl*4 sequence was submitted to GenBank and was assigned the accession number HM125534. *In silico* protein translation analyses revealed that LYZL4 is encoded by four exons (Figure 2), which is in agreement with the predicted *Lyzl*4 sequence available at GenBank. It is thought to be secretory because of the presence of a signal peptide. The predicted physical characteristics of the rat LYZL proteins are given in Table 1. An important feature is that all the rat LYZL proteins retained the characteristic eight cysteine signature of c-type lysozymes (Figure 3A). In LYZL4 one of the essential amino acids (aspartate) of c-type lysozyme active site is replaced by glycine (Figure 3A). Similarly, in LYZL3, aspartate is replaced by aspargine, suggesting that LYZL3 and LYZL4 may not exhibit lysozyme activity. However, the essential amino acids of the active site are conserved in LYZL1 and LYZL6 (Figure 3A). Loss of aspartate in the lysozyme active site was also observed in the human and mouse LYZL4 (Figure 3B). Sequence analyses reveal that the rat LYZL proteins are highly homologous to their mouse and human counterparts (Table 1). Similarly, based on the ClustalW2 score, the homology among the rat LYZL proteins was also found to be high (Table 2). Rat LYZL4 displays a high degree of homology with mouse SLLP1 (Figure 3C). Homology modeling using mouse SLLP1 as the template was carried out to determine the three dimensional structure of rat LYZL4 (Figure 4A and 4B). LYZL4 seems to be structurally similar to the mouse SLLP1 except at few residues as shown in the superimposed image (Figure 4C). There are 7 helices and 4 disulphide bridges that are conserved between the mouse SLLP1 and rat LYZL4. The beta sheets present between residues 43–60 in mouse counterpart are absent in the rat LYZL4. A change in secondary structure pattern was also observed near residue 23 wherein a helix is formed in case of mouse SLLP1 and not in rat LYZL4. According to Ramachandran plot, 90.8% of the residues lie in the most favored regions and 9.2% in the additionally allowed regions (Figure 4D). There are no disallowed regions predicted. The generated model seems to be reliable with the good Ramachandran plot values with a G-factor of -0.12 (Figure 4D). The RMSD value was 0.405 which was within the agreeable limit. The similarity in structural and tissue localization of rat LYZL4 and mouse SLLP1 suggests that they may exhibit similar function.

**Table 1:** General characteristic features of rat lysozyme-like proteins.

	LYZL1	LYZL3	LYZL4	LYZL6
**Amino acids**	148	163	145	148
**Molecular weight**	16589.9	18303.7	16304.8	17027.6
**Signal peptide**	1-19	1-35	1-19	1-19
**pI**	8.38	6.4	5.79	5.74
**Active site**	Conserved(Glu-54 & Asp 71)	NIL	NIL	Conserved(Glu-54 & Asp 71)
**Localization**	Secretory	Secretory	Secretory	Secretory
**Homology with human counterpart**	86%	89%	86%	84%
**Homology with mouse counterpart**	95%	95%	97%	93%
**Amino acids involved in disulfide bonds**	25 & 145, 49 & 133, 83 & 98,94 & 112	41 & 161, 65 & 149, 99 &114,110 & 128	25 & 143, 49 & 130, 84 &95, 91 & 109	25 & 145, 49 & 133, 83 & 98,94 & 112
**Myristylation sites**	NIL	NIL	NIL	NIL
**O-glycosylation sites**	NIL	NIL	NIL	NIL
**Phosphorylation sites**	19, 55, 135, 138, 59, 102, 53, 57, 72	32, 39	70, 133, 63	88,81,86

**Table 2:** Homology (ClustalW2 score) between rat LYZL proteins.

	Rat-Lysozyme	LYZL1	LYZL3	LYZL4	LYZL6
Rat Lysozyme		41	38	36	37
LYZL1			41	42	37
LYZL3				43	37
LYZL4					46
LYZL6					

### *Lyzl* gene expression in the rat

**Figure 5:**
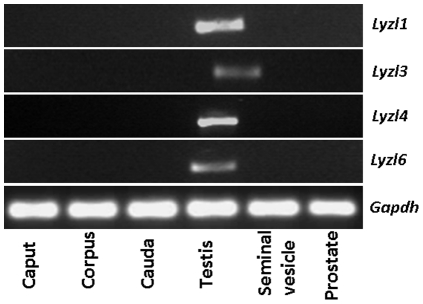
*Lyzl* expression in the rat male reproductive tract. Total RNA isolated from caput, corpus, cauda, testis, seminal vesicle and prostate were reverse transcribed and PCR amplified.

**Figure 6:**
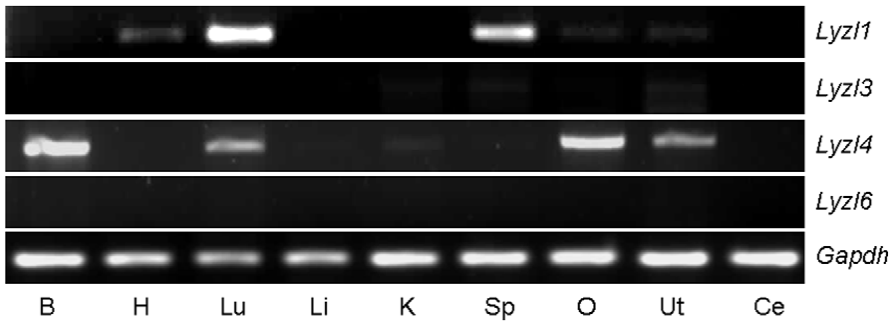
Tissue distribution of *Lyzl1, 3, 4* and *Lyzl6* in the rat. RT-PCR analysis was performed using total RNA isolated from **B**rain, **H**eart, **L**ung, **L**iver, **K**idney, **Sp**leen, **O**vary, **Ut**erus and **C**ervix.

Using RT-PCR analyses, the expression pattern of rat *Lyzl* genes was determined in the male reproductive tract. All the *Lyzl* genes analyzed in this study were found to be expressed specifically in the testes (Figure 5). To determine if the expression pattern of *Lyzl* mRNA transcripts is male reproductive tract specific, RT-PCR was performed in a variety of tissues obtained from male and female rats. *Lyzl1* was found to be expressed in the heart, lung and spleen, whereas *Lyzl*4 was found to be expressed in the brain, lung, ovary and uterus (Figure 6). *Lyzl3* and *Lyzl6* expression was not found in these tissues suggesting that their expression is highly male reproductive tract specific (Figure 6).

### Androgen dependent expression

**Figure 7:**
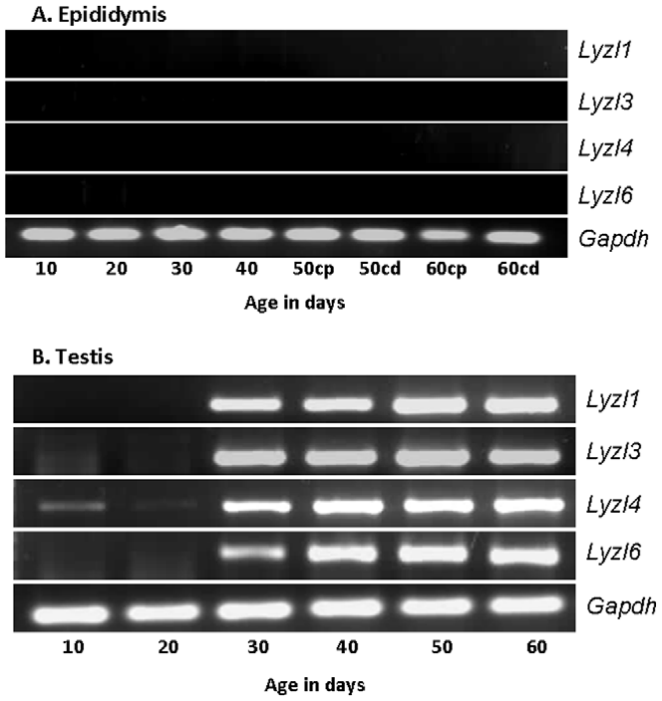
Developmental expression pattern of *Lyzl* transcripts in the epididymides and testes of rats. RT-PCR for *Lyzl1, 3, 4* and *Lyzl6* was performed using RNA isolated from the epididymides and testes of 10–60 day old rats.

Gene expression in the male reproductive tract is under the influence of androgens [21], [22]. To elucidate the influence of androgen variation, PCR analyses for *Lyzl*s were carried out using total RNA isolated from the epididymides and testes of 10–60 day old rats. Though the expression of *Lyzl* transcripts is absent in the epididymis obtained from the adult rats (Figure 5), it is possible that they may be expressed in the younger rats during postnatal development. In the epididymis, none of the *Lyzl*s analyzed in this study were expressed at all the ages during development (Figure 7A). In the testes, *Lyzl1*, 3 and 6 were expressed starting from day 30 during postnatal development, whereas *Lyzl*4 was expressed in all the age groups (Figure 7B).

### Immunolocalization

**Figure 8:**
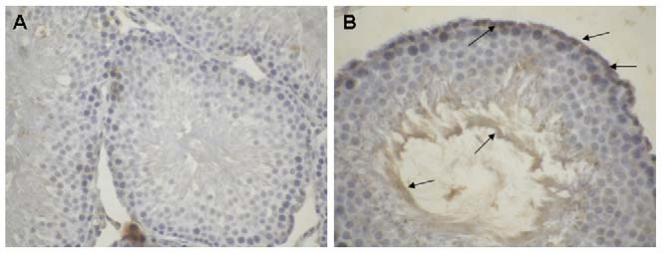
Immunolocalization of rat LYZL4 in the rat testis. Serial sections of the rat testis were incubated with antigen preadsorbed (A) and LYZL4 polyclonal antibody (B). Magnification – 40X.

**Figure 9:**
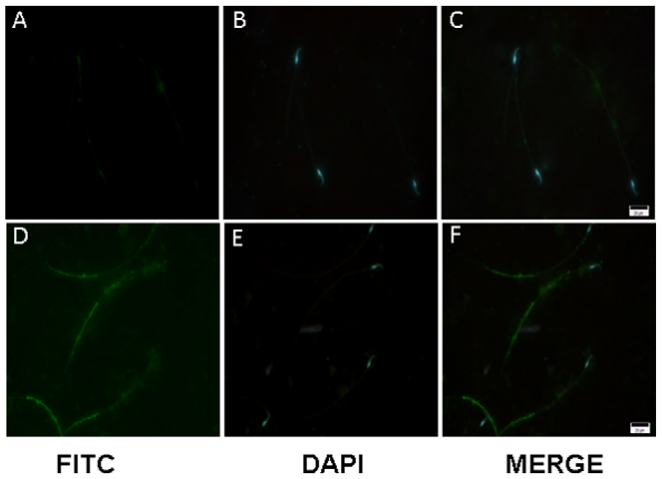
Immunofluorescence detection of LYZL4 on the rat sperm. **A–C**, Immunofluorescent staining using antibody preincubated with antigen. **D–F**, Immunofluorescent staining with LYZL4 antibody alone.

Since *Lyzl*4 was the only one predicted among the *Lyzl*s analyzed in this study and that its expression was found to be present during all stages of postnatal development, we further studied its expression pattern at the protein level in the testis using polyclonal antibodies raised against LYZL4. It was found to be expressed in the germinal epithelium and concentrated on the developing spermatozoa (Figure 8). Using immunofluorescence, we observed that LYZL4 is expressed only in the tail region of the sperm obtained from adult rat (Figure 9), suggesting that LYZL4 may contribute to the motility of the spermatozoa.

### Muramidase, isopeptidase and antimicrobial activities

**Figure 10:**
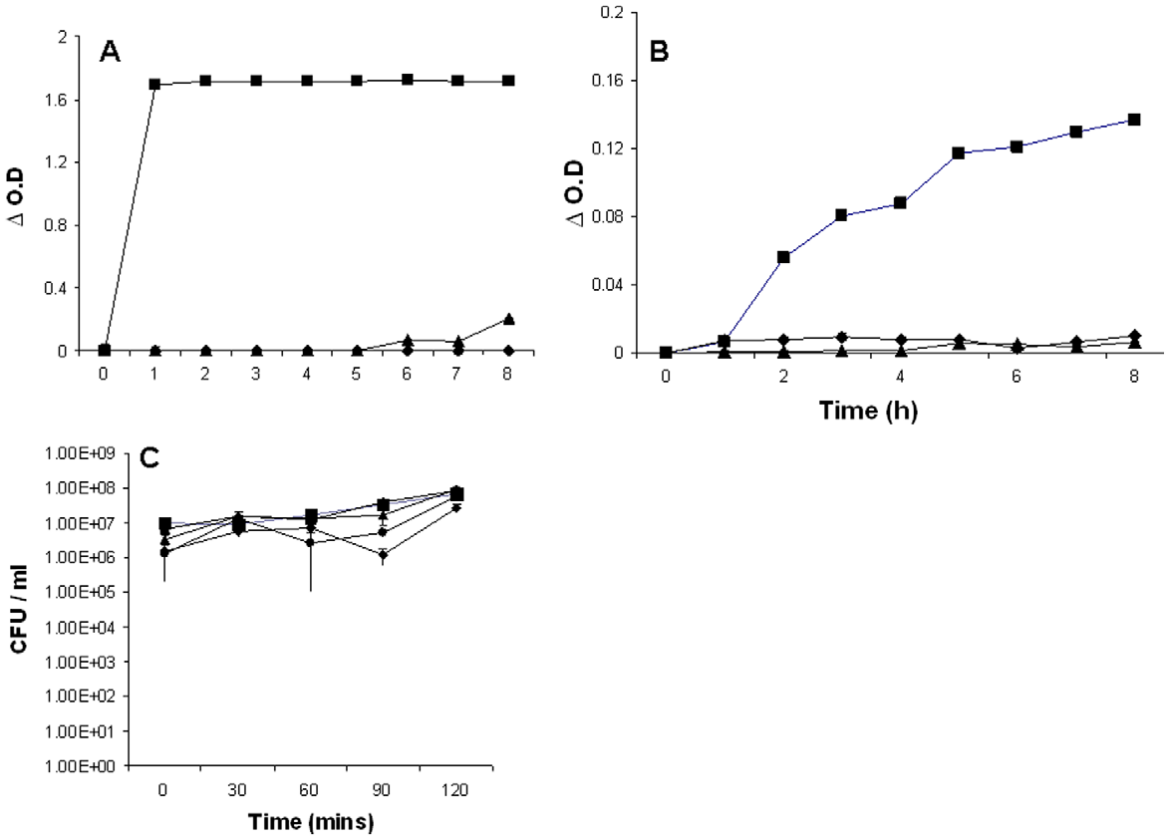
Muramidase, isopeptidase and antibacterial activities of rat LYZL4. **A, B**) 1 (▴) and 5 (♦) µM rat recombinant LYZL4 protein was incubated at 37°C and the formation of product due to muramidase and isopeptidase activities was measured spectrophotometrically at 450 and 405 nm respectively. 1.71 µM (25 µg/ml) lysozyme (▪) was used as a positive control. **C**) Mid-log phase *E. coli* were incubated with 0 (▪), 10 (▾), 25 (▴), 50 (•) and 100 (♦) µg/ml rat recombinant LYZL4 protein for 0–120 min. Values shown are Mean ± S.D.

LYZL4 being a c-type lysozyme is expected to exhibit the hydrolytic activity of glycosyl bonds. Hence, its muramidase and isopeptidase activities were analyzed. At the concentrations tested (1 and 5 µM) no activity was displayed by LYZL4 (Figure 10A and 10B). The positive control, lysozyme, displayed potent muramidase and isopeptidase activities. The antimicrobial activity of lysozyme is well known. To determine whether recombinant rat LYZL4 protein exhibits antimicrobial activity, its ability to kill *E. coli* was tested using colony forming unit (CFU) assay. LYZL4 (10–100 µg/ml) did not display any antibacterial activity at all the concentrations tested (Figure 10C). The negative control, LCN6, an epididymal lipocalin, did not show any detectable antibacterial activity when incubated for 2 h at concentrations up to 100* µ*g/ml (data not shown).

## Discussion

C-type lysozyme is expressed in most species and because of its ability to act on microbial membranes, it is thought to play an important role in innate immune defense. In the recent years, lysozyme-like proteins were identified and characterized in the human and mouse [17], [18], [20]; their expression being reported in the male reproductive tract. The mRNA and protein expression pattern of human and mouse LYZLs varied within the male reproductive tract, suggesting that they may play different roles in these two species [17], [18], [20]. However, the expression pattern of lysozyme-like genes and proteins are not characterized in the rat. In this study, we analyzed their mRNA and LYZL4 protein expression to determine whether rat lysozyme-like gene function is similar to that of human and mouse.

In our rat genome mining, we identified four *Lyzl* genes distributed on chromosomes 8 (*Lyzl4*), 10 (*Lyzl3* and 6) and 17 (*Lyzl1*). Basing on the accession number, *Lyzl4* was found to be predicted. However, we did not find any literature that describes the identification and characterization of rat *Lyzl1*, 3 and 6, though they are shown as reported in GenBank. Hence, we submitted only the sequence of *Lyzl4* to Genbank. *In silico* analyses revealed that *Lyzl4* present on chromosome 8 is encoded by 4 exons, which is in agreement with the gene structure available at GenBank. Its homology with its human and mouse counter parts suggests that LYZL4 is highly conserved. The four rat *Lyzl* genes are distributed on three chromosomes. Such distribution of *Lyzl* genes on three chromosomes was also observed in human and mouse, indicating a possible organizational conservation. Homology between the rat LYZL proteins suggests that they might have originated from a common progenitor and may share a common physiological function. The presence of eight cysteine signature in the rat LYZLs supports the classification of these to the c-type lysozyme family. Homology modeling reveals a close structural similarity of rat LYZL4 with mouse SLLP1. Such structural similarity suggests conservation of c-type lysozyme like proteins between the species.

To the best of our knowledge, we report for the first time the expression pattern of *Lyzl* mRNA transcripts in the rat. Testis specific expression of *Lyzl6* and *Lyzl3* observed in this study implies a potential role for these genes in testicular function. Though *Lyzl1*, *3* and *4* were expressed in other tissues, their expression was confined to the testis in the male reproductive tract. Such specific mRNA expression was previously reported for other *Lyzl* genes. *Lyzl4* expression in the mouse was found to be testis specific [20]. Similarly, the human *SLLP1* was found to be expressed specifically in the testis [18], whereas the other three human c-type lysozyme like genes identified were expressed in the testis/epididymides [17]. Reproductive tract specific expression was also demonstrated for other genes such as *Spag11e* [23], *DEFB118* [24] and members of the HE2 family [13]. Contrary to the expression pattern of *Lyzl* transcripts of human, the expression of rat *Lyzl* genes in non-reproductive and female reproductive tissues suggests that they may have functions beyond male reproductive tract physiology. Similarly, the expression of *Lyzl2* and *3* mRNA transcripts in the mouse was also found to be present in other tissues beyond the reproductive tract. Results of this study and others reported earlier indicate that variability in tissue specific mRNA expression could contribute to the varied functional role of *Lyzl* genes in different species.

Developmental regulation of a wide variety of genes due to the fluctuations of androgens at various stages in the male reproductive system has been studied extensively [25]. Androgen levels in the rat epididymis decline from birth until 20 days but remain at a substantial level of approximately 10 ng/g tissue (35 nM) until approximately 40 days when the levels begin to increase to that of the adult, between 15–20 ng/g [26]. Serum testosterone levels in the young rat remain low and do not begin to increase to adult levels until 35–40 days of age [27]. Absence of *Lyzl* transcripts in the epididymides obtained from 20–60 day old rats, suggests that their expression pattern is not androgen dependent in this organ system. Testicular androgen variation during development in the rat was reported to be significantly different from the epididymis. A steady increase in testosterone levels occurs in the rete testis of 30-130 day old rats [28], [29]. In this study, the presence of *Lyzl1*, *3* and *6* mRNA transcripts was observed in the testes starting from 30 day post natal development, whereas *Lyzl4* was expressed in all the age groups, though minimally during 10-30 days. The expression pattern of *Lyzl* transcripts analysed in this study seem to correlate with the minimal androgen levels from day 20 to day 40 and increased androgen in the adult [26], suggesting that *Lyzl* expression may be androgen dependent during development in the testis. Androgen dependent expression of *Lyzl4* during development was reported in the mouse [20]. Further studies are required to determine the molecular mechanisms that operate in controlling the expression of *Lyzl* transcripts during development.

To demonstrate whether *Lyzl4* mRNA expression correlates with the protein expression, immunohistochemistry was performed on testicular sections. LYZL4 protein expression in the testes was observed in the germinal epithelium and on the maturing spermatozoa. It is possible that LYZL4 secreted into the lumen could bind to the sperm and aid in their development. Region specific gene expression of a wide variety of testicular and epididymal proteins on the sperm are reported [21]. The presence of LYZL4 specifically on the sperm tail suggests that it is involved in contributing to sperm motility. However, it is intriguing to note that though it is not expressed in the epididymis it is localized on the sperm tail. It is possible that LYZL4 is added on to the surface in the testis and this protein may continue to be present in the tail region in the epididymis.

The catalytic mechanism of c-type lysozymes involves the interaction of Glu-35 and Asp-52 of the active site with beta-1,4 glycosidic bond of the substrate. In this study, rat LYZL4 did not exhibit any muramidase and isopeptidase activity at the concentrations tested. This could be due to the replacement of aspartate by glycine in the catalytic site. Such loss of activity due to “changed” amino acids was reported for human SLLP1 and mouse LYZL4 [18], [20] Epididymal proteins secreted into the lumen play a key role in sperm maturation. Besides this, some of them are known to exhibit potent antimicrobial activity, thereby forming important components of male reproductive tract innate immunity. Lysozyme, because of its ability to cleave the glycosydic bond of peptidoglycan, displays potent antimicrobial activity. In this study, we demonstrate that LYZL4 did not display any antibacterial activity against *E. coli*. The human c-type lysozyme like SLLP1, was non-bacteriolytic similar to the lack of antibacterial activity of rat LYZL4 observed in this study. The inability of rat LYZL4 to exhibit bacterial killing could be due to the modification in its active site.

In conclusion, for the first time, we report the identification of rat Lyzl4 and the expression pattern of Lyzl1, 3 and 6. In the male reproductive tract, their expression was confined to the testes. Lyzl expression seems to be androgen dependent in the testes. Immunolocalisation revealed that Lyzl4 mRNA is translated and the protein is localized on the germinal epithelium and on the sperm tail. LYZL4 did not exhibit antibacterial, muramidase and isopeptidase activities. Results of our study indicate that LYZL4 may play a crucial role in the testis and may also contribute to the motility of the sperm. Further studies are required to demonstrate the molecular mechanisms by which LYZL4 may contribute to these functions.

## Materials and Methods

### In silico analyses

Gene and protein notation used in this study was based on HUGO nomenclature. Gene symbols are italicised, with only the first letter in uppercase and the remaining letters in lowercase (*Lyzl*). Protein designations are the same as the gene symbol, but are not italicised, all uppercase letters (LYZL). The rat *Lyzl4* predicted sequence and *Lyzl1, Lyzl3* and *Lyzl6* sequences were obtained from the rat genome (build RGSC v3.4) at the NCBI website (http://www.ncbi.nlm.nih.gov/). Gene specific primers were designed for each *Lyzl* mRNA (Table 3). RT-PCR was performed using rat testis mRNA as the template. The *Lyzl4* PCR amplicons were sequenced, aligned and deposited in GenBank. The corresponding exon/intron boundaries were determined by aligning the cDNA with the genomic sequence. The sequences were translated and the predicted physical features of the deduced amino acid sequences were analyzed using tools available at ExPASy proteomics server (http://ca.expasy.org/).

**Table 3:** Gene specific primers used in this study.

Gene	Direction	Sequence
***Lyzl*** **1**	Forward	5′-TGTCGG TGT CTT CGC CCT AAT T-3′
	Reverse	5′-GAC GAG TCT TTG CTC TCA CAG T-3′
***Lyzl*** **3**	Forward	5′-TCC AGC AAG GCC AAG GTC TTC A-3′
	Reverse	5′-TAG AAG TCA CAG CCA TCC ACC CA-3′
***Lyzl*** **4**	Forward 1	5′-ATG TGG GCA CTG TTG ACA CCA -3′
	Reverse 1	5′-CTA CAC CAT TGA TCC TGC TCC A-3′
	Forward 2	5′-GTG GTG ATT GAG GAT TCC TTC AG-3′
	Reverse 2	5′-ATG GAG GCA CCA ATC GGA GTC A-3′
	Forward 3	5′-ATG CAG CTG TAC CTG GTG CTT CT-3′
	Reverse 3	5′-GCT GGTTTATTCTGCACCTTGTAC C-3′
***Lyzl*** **6**	Forward	5′-TAT CTG TGT GGT GAG CTG CCT TCT-3′
	Reverse	5′-TGC ACA GTG GAT GGA TGGAAT GAG -3′

The rat lysozyme like 4 (LYZL4) protein structure was predicted by homology modeling using MODELLER9v5. Basing on the BLAST search against PDB, the mouse sperm c-type lysozyme like protein 1 (SLLP1; PDB code 2GOI) was chosen as template because of its sequence similarity. The reliability of modeled structure was validated by Ramachandran plot analyses using PROCHECK and the correlation in structure between the template and model was verified by analyzing the RMSD values using PyMOL.

### Tissue specimens and RT-PCR

Wistar rats (aged 60–90 days; n = 3) were obtained from National Institute of Nutrition, Hyderabad, India. Tissues collected were placed in RNA*Later* (Ambion Inc, Austin, TX, USA) solution overnight at 4°C to allow penetration and fixation and stored at -70°C. Total RNA was extracted using the TRIzol reagent (Invitrogen, Carlsbad, CA, USA) from the following tissues: the three regions of the epididymis (caput, corpus and cauda), testis, prostate, seminal vesicle, brain, liver, lung, kidney, heart, spleen, cervix, ovary and uterus. Total RNA (2 µg) was reverse transcribed using 200 U SuperSciptIII (Invitrogen, Carlsbad, CA, USA) and 0.5 µg of oligodT (Invitrogen, Carlsbad, CA, USA) according to the manufacturer's instructions. 2 µl of the resultant cDNA was amplified by PCR using gene specific primers (Table 1) for *Lyzl1, Lyzl3, Lyzl4, Lyzl6* and *Gapdh*. PCR was performed under the following conditions: 94°C for 2 min followed by 25–35 cycles at 94°C for 30 sec, 56°C for 30 sec and 72°C for 30 sec, and with a final round of extension at 72°C for 10 min. PCR amplicons were analyzed by electrophoresis on 2% agarose gels. For studies on the developmental regulation of *Lyzl* genes, epididymides and testes were collected from 10–60 day old Wistar rats (n = 3) purchased from the National Institute of Nutrition, Hyderabad, India.

### Recombinant protein production

Recombinant LYZL4 protein was prepared as described earlier [30]. Briefly, the open reading frame that corresponds to the rat LYZL4 full length without the signal peptide (amino acid sequence shown in bold in Figure 3) was cloned into pQE30 expression vector (Qiagen, Valencia, CA, USA). *E. coli* (BL-21) was transformed with pQE30 vector containing rat *Lyzl4* cDNA according to the supplier's instructions. Fusion protein expression was induced with 1 mM isopropyl-1-thio-β-D-galactoside for 3 h at 37°C. 1% glucose was maintained in the medium to avoid baseline expression of the protein prior to induction. Bacterial lysate incubated with nickel-nitrilotriacetic acid-agarose (Qiagen) for 1 h to allow binding of His-tagged recombinant protein to the resin, was then transferred to a column, washed and eluted according to the manufacturer's recommendations. The His-tagged recombinant LYZL4 protein contained the following additional amino acid residues at the N-terminus (MRGSHHHHHHGS) due to the construction of the vector. Fractions were analyzed on 15% gradient polyacrylamide Tris-Tricine gels and stained with Coomassie blue G250. Further, the identity of the protein was confirmed by Western blotting using anti-His-tag antibody. Fractions containing purified protein were pooled and dialyzed against phosphate buffered saline (pH 7.4) to remove urea.

### Antibody production and immunodetection

Antibodies to detect rat LYZL4 were raised in our laboratory. Briefly, rabbits were immunized with recombinant LYZL4 protein mixed with complete adjuvant followed by booster doses 4 and 6 weeks after initial immunization. Antiserum was collected 2 weeks after the second booster dose. For immunohistochemical staining, testes were fixed in Bouin's fluid and embedded in paraffin. Five micron thick sections were taken and treated with xylene and graded alcohol (70-100%). The sections were then treated with 1% Triton-X 100 to facilitate permeabilisation followed by treatment with 3% H_2_O_2_. LYZL4 was detected by incubating the sections using polyclonal antibodies (1∶250 dilution) raised in rabbit followed by biotin conjugated secondary antibody (1∶500 dilution) against rabbit IgG raised in goat. Immunostaining was detected using a Vectastain Elite ABC kit (avidin- biotin-complex horse radish peroxidase) (Vector Laboratories Inc., Burlingame, USA). Diaminobenzidine, the chromogen, produced a brown reaction product. Sections were counter-stained with hematoxylin. For the control staining, antibodies were preincubated with antigen (LYZL4 recombinant protein). Immunofluorescence on the sperm was detected by using anti-rabbit secondary antibodies tagged with FITC. Photographs were taken using a color digital imaging system attached to a Leica Photomicroscope. Surgical procedures were conducted using the guidelines for the care and use of laboratory animals and this study was specifically approved by the Institutional Animal Ethics Committee of University of Hyderabad (LS/IAEC/YS/11/07).

### Antibacterial assay

Colony forming units (CFU) assay was employed to test the antibacterial activity as described previously [30]. Briefly, overnight cultures of *E*. *coli* XL-1 Blue (Stratagene, La Jolla, CA, USA) were allowed to grow to mid-log phase (*A*_600_  =  0.4 – 0.5) and diluted with 10 mM sodium phosphate buffer (pH 7.4). Approximately 2×10^6^ CFU/ml of bacteria were incubated at 37°C with 10–100 µg/ml of the LYZL4 recombinant protein and aliquots of the assay mixture were taken at 30, 60, 90 and 120 min after the start of incubation. After incubation, the assay mixtures were serially diluted with 10 mM sodium phosphate buffer (pH 7.4) and 100 µl of each was spread on a Luria–Bertani agar plate and incubated at 37°C overnight to allow full colony development. The resulting colonies were hand counted and plotted as log CFU/ml. As a negative control, the epididymal lipocalin, LCN6 (recombinant protein expressed and purified using the same procedure followed for LYZL4) was used in the assays. Values shown are Mean ± S.D. Statistical analyses were performed using Sigma plot software.

### Muramidase (lysozyme) assay

The muramidase activity of recombinant rat LYZL4 was determined using a standard protocol described earlier [6]. Briefly, rat LYZL4 (1 µM and 5 µM) was incubated with 2 ml of *M. lysodeikticus* (Sigma Aldrich, USA) cells in 50 mM KH_2_PO_4_-NaOH buffer, pH 7.0 and the decrease in turbidity was monitored at 450 nm in a spectrophotometer.

### Isopeptidase assay

The isopeptidase activity of recombinant rat LYZL4 was measured according to the method described earlier [6], wherein, the substrate, L-γ-glutamine-p-nitroanilide (L- γ -Glu-pNA) is cleaved to produce p-nitroanilide (pNA), which can be detected at 405 nm. Rat LYZL4 (1 µM and 5 µM) was added to the reaction mixture containing 1.75 mM substrate in 0.05 M MOPS buffer. The formation of pNA was monitored spectrophotometrically at 405 nm.
